# High-throughput sequencing data revealed genotype-specific changes evoked by heat stress in crown tissue of barley *sdw1* near-isogenic lines

**DOI:** 10.1186/s12864-022-08410-1

**Published:** 2022-03-04

**Authors:** Krzysztof Mikołajczak, Anetta Kuczyńska, Piotr Ogrodowicz, Agnieszka Kiełbowicz-Matuk, Hanna Ćwiek-Kupczyńska, Agata Daszkowska-Golec, Iwona Szarejko, Maria Surma, Paweł Krajewski

**Affiliations:** 1 Institute of Plant Genetics, Polish Academy of Sciences, Poznań, Poland; 2 Institute of Biology, Biotechnology and Environmental Protection, Faculty of Natural Sciences, University of Silesia in Katowice, Katowice, Poland

**Keywords:** Crown tissue, Gibberellin-related genes, Response to temperature, *Hordeum vulgare* L., RNA-seq, Single nucleotide polymorphism

## Abstract

**Background:**

High temperature shock is becoming increasingly common in our climate, affecting plant growth and productivity. The ability of a plant to survive stress is a complex phenomenon. One of the essential tissues for plant performance under various environmental stimuli is the crown. However, the molecular characterization of this region remains poorly investigated. Gibberellins play a fundamental role in whole-plant stature formation. This study identified plant stature modifications and crown-specific transcriptome re-modeling in gibberellin-deficient barley *sdw1*.*a* (BW827) and *sdw1.d* (BW828) mutants exposed to increased temperature.

**Results:**

The deletion around the *sdw1* gene in BW827 was found to encompass at least 13 genes with primarily regulatory functions. A bigger genetic polymorphism of BW828 than of BW827 in relation to wild type was revealed. Transcriptome-wide sequencing (RNA-seq) revealed several differentially expressed genes involved in gibberellin metabolism and heat response located outside of introgression regions. It was found that *HvGA20ox4*, a paralogue of the *HvGA20ox2* gene, was upregulated in BW828 relative to other genotypes, which manifested as basal internode elongation. The transcriptome response to elevated temperature differed in the crown of *sdw1.a* and *sdw1.d* mutants; it was most contrasting for *HvHsf* genes upregulated under elevated temperature in BW828, whereas those specific to BW827 were downregulated. In-depth examination of *sdw1* mutants revealed also some differences in their phenotypes and physiology.

**Conclusions:**

We concluded that despite the studied *sdw1* mutants being genetically related, their heat response seemed to be genotype-specific and observed differences resulted from genetic background diversity rather than single gene mutation, multiple gene deletion, or allele-specific expression of the *HvGA20ox2* gene. Differences in the expressional reaction of genes to heat in different *sdw1* mutants, found to be independent of the polymorphism, could be further explained by in-depth studies of the regulatory factors acting in the studied system. Our findings are particularly important in genetic research area since molecular response of crown tissue has been marginally investigated, and can be useful for wide genetic research of crops since barley has become a model plant for them.

**Supplementary Information:**

The online version contains supplementary material available at 10.1186/s12864-022-08410-1.

## Background

Global warming increases the prevalence of high-temperature stress in plants [1]. Heat shock disrupts biological processes and damages membranes, cellular components, and overall organization, limiting plant growth and productivity [2]. Alternations in plant metabolism underlying plant adaptation to heat stress have been increasingly explored by researchers [3, 4]. However, the mechanisms of the molecular responses to elevated temperatures are far from being fully understood. Heat shock proteins (HSPs) and heat shock transcription factors (HSFs) play a central role in plant defense against temperature stress. HSPs are divided into five major size classes: HSP100, HSP90, HSP70, HSP60, and small HSPs [5]. HSPs contribute to a series of processes under stressful and optimum conditions, protecting other proteins against heat-induced denaturation [6]. HSFs regulate HSP-mediated responses to temperature stress. Three structural classes (A, B, and C) of *HSF* genes have been identified in model plants [7]. Class A is suggested to be responsible for regulating the transcription of heat-responsive genes by binding to the heat shock elements of target genes [8]. HSFs belonging to class B are supposed to be repressors of stress-inducible genes, including other HSF- and HSP-coding genes, during permanent heat exposure [9]. Finally, class C is thought to be involved in abscisic acid-mediated responses to abiotic stresses at reproductive stages [10].

Barley (*Hordeum vulgare* L.) is one of the most important cereal crops worldwide (faostat.fao.org). Its genome has been sequenced [11], and it represents a convenient genetic model for *Triticeae* research [12]. Numerous studies have focused on various aspects of barley plant development in the context of canopy elements, such as the characteristics of tillers or plant height [13, 14]. However, the crown (first node above the seed), an essential tissue for plant performance under various environmental stimuli, remains poorly investigated. In cereals, the regeneration of roots and shoots after exposure to stress is controlled by meristems located in crowns. Thus, the ability of a plant to survive stress depends on the viability of its crown tissues [15, 16]. A growing point located in the crown tissue regulates shoot branching [16]. Although tillering occurs during early vegetative growth, it is considered an important target for manipulating plant architecture [17], as it determines the final plant stature and grain yield.

The molecular characterization of the crown region is marginally known compared with that of other above-ground crop organs, especially leaves. Available reports have focused mainly on wheat [18]. Small-scale molecular studies aimed at characterizing drought-induced modifications of the crown proteome in barley [16] using 2D-gel electrophoresis and tandem mass spectrometry identified differential abundance of several proteins involved in energy metabolism and protein degradation. In another study [19] microarrays were employed to compare gene expression in the leaves and crowns of winter barley and authors concluded that the crown, responding specifically to cold stress, plays a crucial role in plant survival.

In monocots, molecular mechanisms regulating tiller development and plant stature involving the crosstalk among genetic, hormonal networks, and environmental factors remain to be unraveled. Gibberellins (GAs) play a fundamental role in whole-plant stature formation [20]. GA-20-oxidases (*GA20ox*), GA-3-oxidases (*GA3ox*), and GA-2-oxidases (*GA2ox*) are crucial enzymes responsible for gibberellin homeostasis [20–22]. Any functional disorder in essential GA biosynthesis enzymes affects plant stature [23]. A loss of function of *GA20ox* or *GA3ox* decreases GA levels leading to reduced plant height, whereas overexpression stimulates growth. In contrast, increased *GA2ox* expression causes a dwarf phenotype by decreasing internode elongation [23–26]. A well-known GA biosynthesis gene in barley is *sdw1*/*denso*. Four barley mutants carrying *sdw1* are known: a spontaneous mutant *sdw1.c* selected from the ‘Abed Denso’ variety and three forms obtained using physical mutagens: *sdw1.a* from the ‘Jotun’ variety, *sdw1.d* from the ‘Valticky’ variety (released as variety ‘Diamant’), and *sdw1.e* from the variety ‘Bomi’ [13, 27]. Short-statured barley genotypes lacking an appropriate *sdw1* function are GA-sensitive and respond to exogenous GAs [28], similar to *sd1*-rice mutants [24, 29, 30]. The *HvGA20ox2* gene encoding gibberellin oxidase has been postulated as a functional gene in the *sdw1* locus [31]. Promisingly, an extensive collection of barley near-isogenic lines (NILs) derived from the ‘Bowman’ cultivar has enabled the evaluation of genetic background effects on plant behavior [32].

This study’s objective was to elucidate plant modifications and crown-specific transcriptome re-modeling that takes place in gibberellin-deficient barley *sdw1* NILs exposed to increased temperatures. Experiments were designed to determine whether the phenotypic expression of *sdw1* mutants is influenced by the allele-specific expression of the *HvGA20ox2* gene or by wider genetic background variance and environmental cues. To achieve this goal, plants were examined considering phenotypic properties, physiological responses, and genomic constitution using high-throughput genotyping and *sdw1* gene sequencing. We also employed the mRNA-seq method to acquire transcriptome-wide characterization of the crown tissue and provide new insights into the expression profiles of heat- and gibberellin-related genes.

## Results

**Fig. 1:**
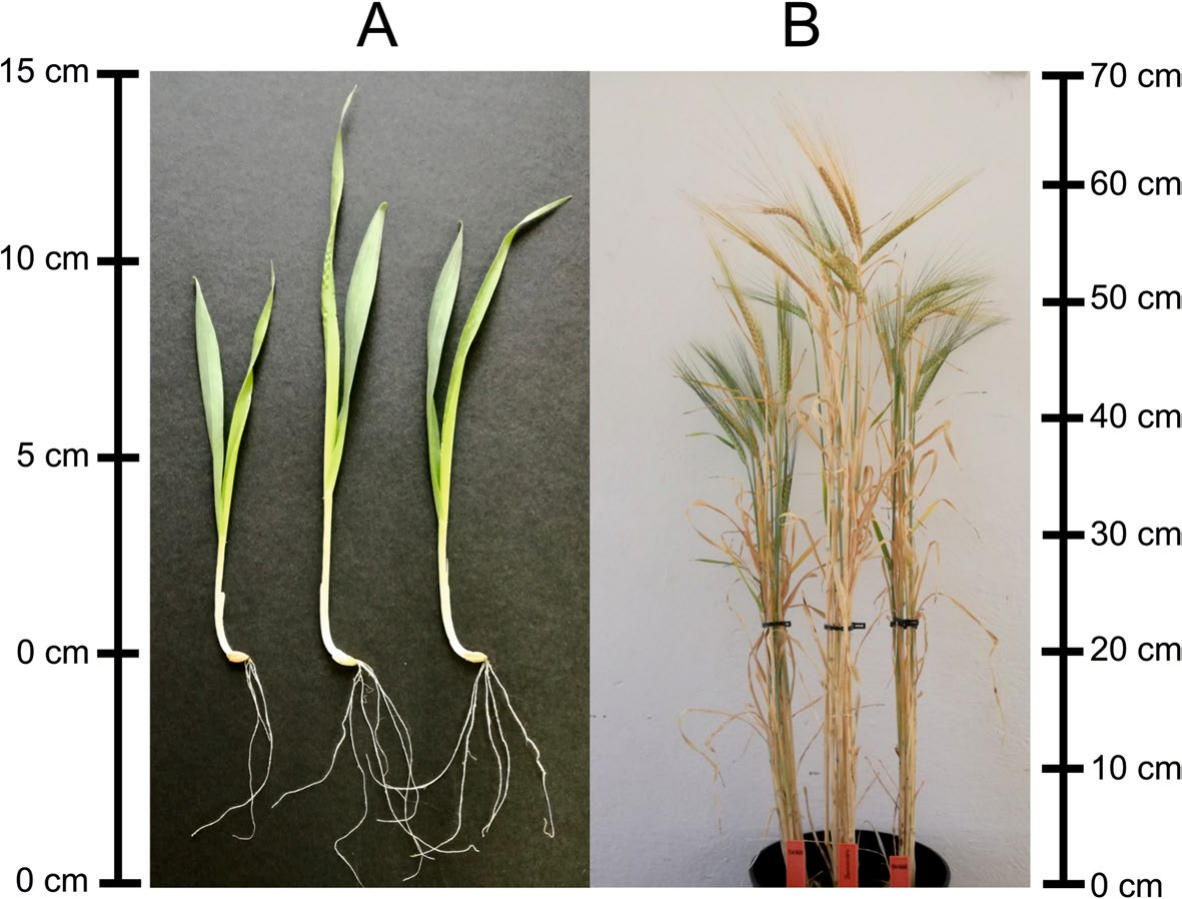
**A** Two-weeks old seedlings, **B** Plants about 80 days after sowing. From left to right: BW827, BW, BW828

Herein, Bowman (BW) and its near isogenic lines BW827 (*sdw1.a*) and BW828 (*sdw1.d*) were examined (Fig. 1).

### *sdw1* gene polymorphism

Sequencing of the *sdw1* gene revealed a 7-bp deletion in BW828 in exon 1 of transcript isoforms 3 and 4 relative to BW. In addition, a G/A SNP at chr3H:634079937 was confirmed (Additional file 1: Fig. S1). Complete deletion of this gene was found in BW827. Based on missing observations obtained for BW827 in all three SNP detection systems and lack of gene expression in this genomic region (see the following sections), it was inferred that a fragment longer than *sdw1*, at least of the region 3H:634071757-634626826, was deleted in this line. This fragment contains 13 genes, according to annotation in IBSC_v2 barley genome version (Ensembl Plants), or 18 genes, as suggested by the newer annotation of the barley pan-genome [33] (Additional file 2: Table S1).

### Single nucleotide polymorphism

**Fig. 2:**
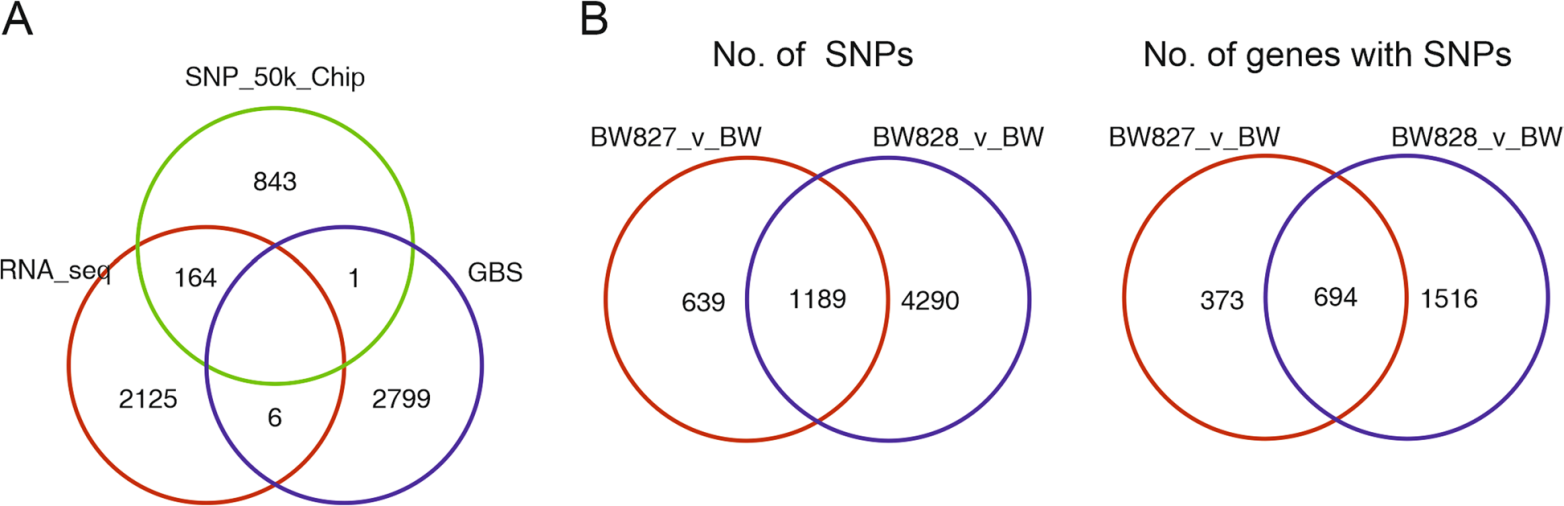
**A** Numbers of SNPs detected by different methods, **B** Numbers of SNPs and genes with SNPs common and specific to polymorphisms between Bowman (BW) and NILs (BW827, BW828)

**Fig. 3:**
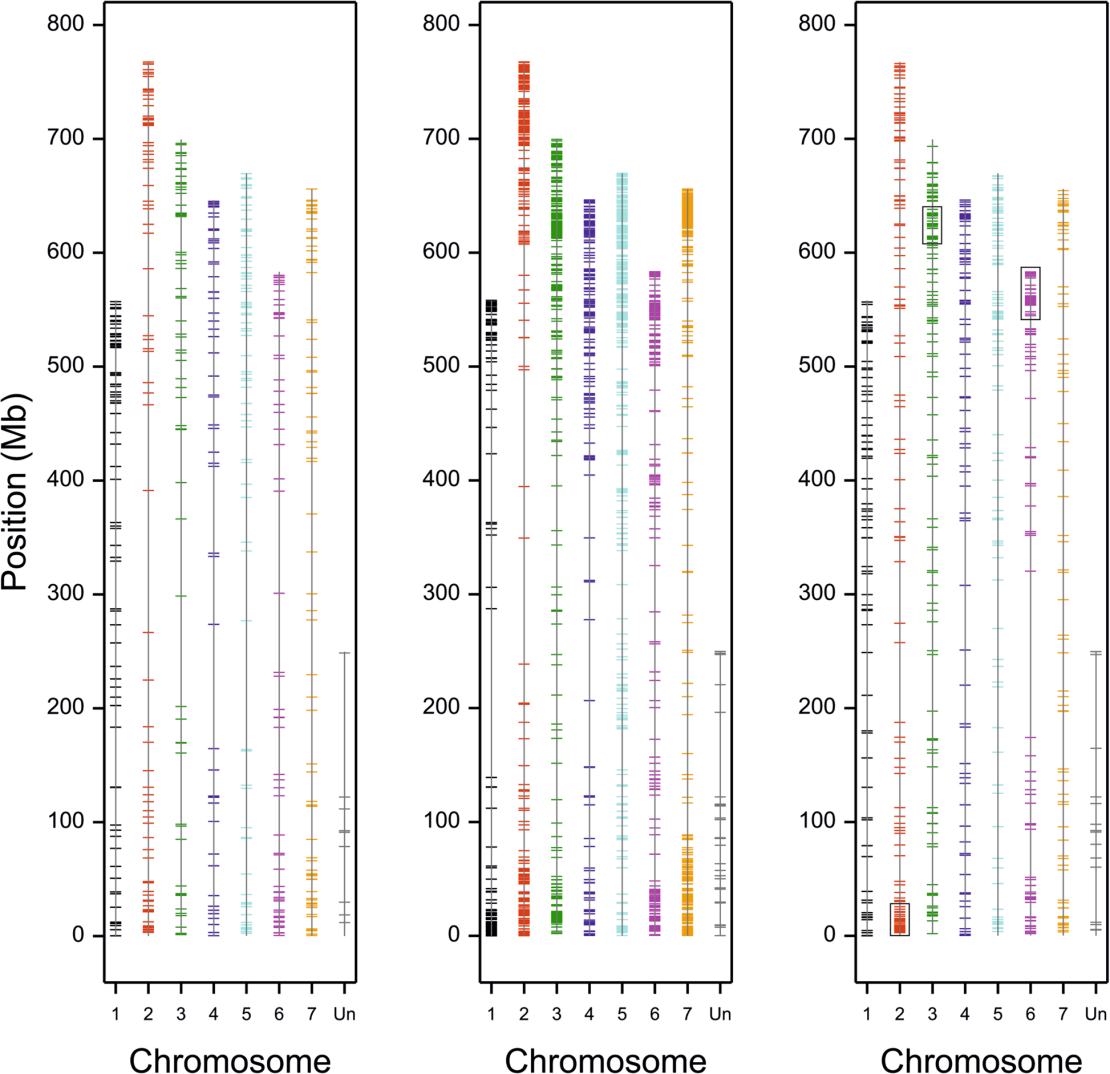
Localization of SNPs observed in NILs relative to Bowman in barley chromosomes. From left to right: SNPs in BW827, SNPs in BW828, SNPs in both NILs. Rectangles mark hotspots with more than 4 SNPs per 1 Mb

The three applied SNP detection methods provided information on 5,938 polymorphic loci between at least two studied barley accessions, with some loci detected by more than one method (Table 1, Fig. 2A, Additional file 3: Table S2). For the 177 SNPs found using two different methods, the readings were generally consistent, except for 6 cases, where pairs of observed SNPs provided different information about differences between genotypes (Additional file 4: Table S3); in all these cases but one the differences could be explained by lack of gene expression or mapping of sequences to different strands of the genome. The fraction of polymorphic loci homozygous in all three genotypes was the largest for the GBS protocol (66.5%) and the lowest for RNA-seq (46.0%) (Table 1). Homozygosity of genotypes was estimated as follows: BW, 81.01%; BW827, 81.97%; and BW828, 82.76%. Similarity to ‘Bowman’ (% of markers with no SNPs between forms) was estimated for BW827 at 70.12% and for BW828 at 10.44%. Out of all SNPs and 2,583 genes that SNPs were assigned to (by the VEP tool), 1189 and 694 were common for NILs, respectively (Fig. 2B). A total of 1684 genes contained one SNP, while the maximum number of SNPs mapped in one gene was 22. Three hotspots for SNPs common to both NILs (with more SNPs than 4 per 1 Mb) were found in the long arm of chromosome 2 (0 – 38 Mb) and the short arms of chromosomes 3 (612 – 646 Mb) and 6 (551 – 580 Mb) (Fig. 3).

**Table 1:** Characteristics of SNP sets obtained by three different protocols; percentage (%) was calculated in relation to the total number of SNPs

Protocol	Number of SNPs	Number of homozygous SNPs (all genotypes homozygous)	Effects predicted by VEP (Ensembl Plants)					Percentage of SNPs with High, Low, or Moderate effects
			HIGH	LOW	MODERATE	MODIFIER	Number of genes with predicted SNP effect	
RNA-seq	2295	1055 (46.0%)	56 (2.54%)	745 (33.82%)	744 (33.77%)	658 (29.87%)	1059	70.13
50k Chip	1017	611 (60.1%)	14 (1.42%)	180 (18.24%)	156 (15.81%)	637 (64.54%)	560	35.46
GBS	2806	1865 (66.5%)	23 (0.83%)	393 (14.24%)	366 (13.27%)	1977 (71.66%)	1268	28.34

Gene Ontology (GO) term analysis of genes with SNPs was performed (Additional file 5: Table S4). The set of 438 polymorphic genes present in the three abovementioned SNP hotspots revealed significant (FDR < 0.05) overrepresentation of the terms ‘chromatin DNA binding’ (5 genes), ‘oxidoreductase activity’ (48 genes), and ‘metal ion binding’ (62 genes).

The fraction of ‘HIGH’, ‘LOW’, or ‘MODERATE’ SNP protein translation effects was the largest for polymorphisms obtained from RNA-seq data (70.13%; Table 1) as expected, due to the data source; however, this implies that 29,87% of RNA polymorphisms were not located in annotated coding sequences and were only assigned the ‘MODIFIER’ status.

### Differential gene expression

**Fig. 4:**
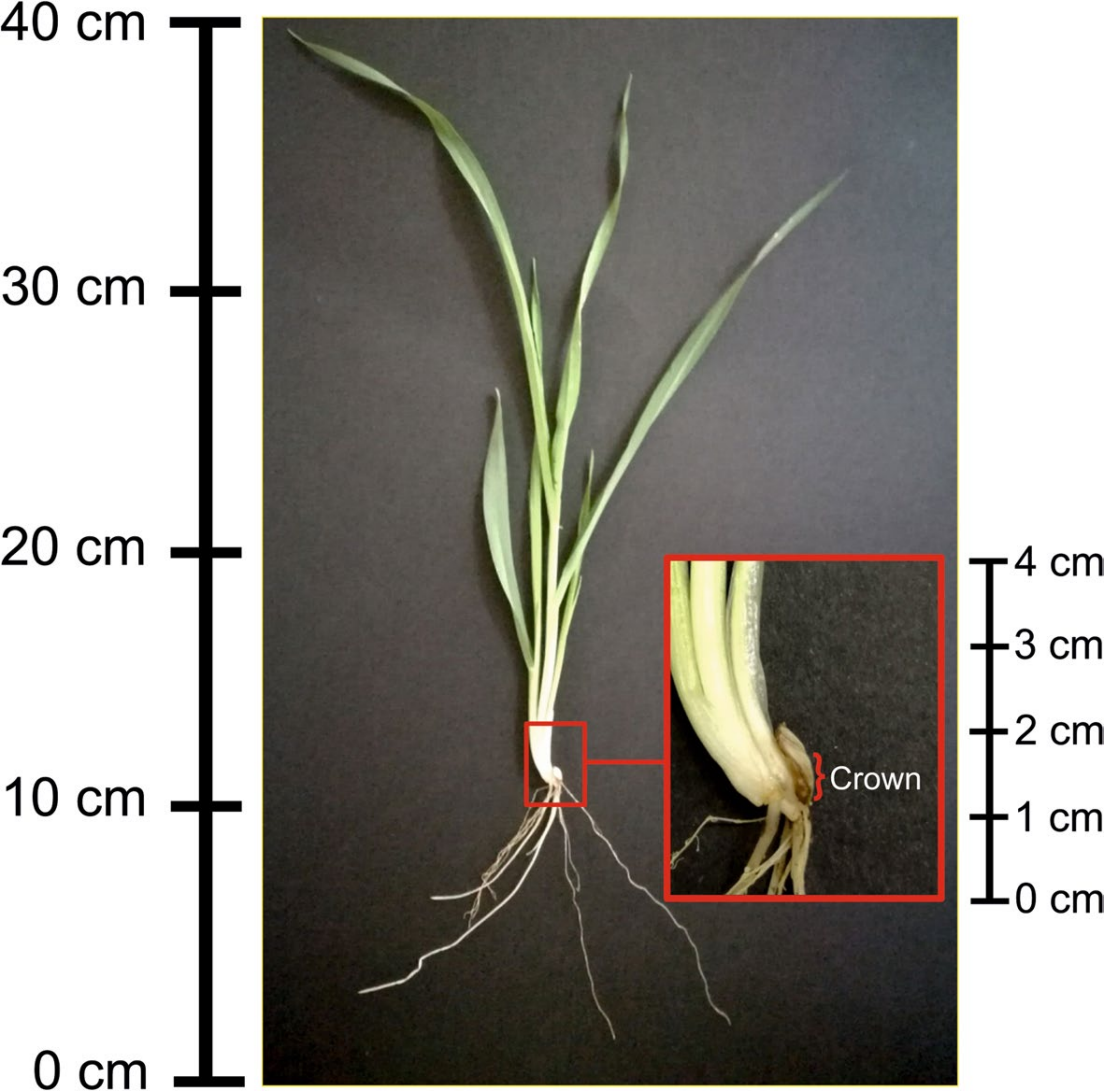
Seedling of cv. Bowman; area marked in red represents the crown region

Crown tissue (Fig. 4) of each genotype was collected for next generation sequencing (NGS). The mean correlation between NGS read counts in biological replications within 12 experimental variants (3 genotypes × 2 treatments × 2 time points) varied from 0.59 to 0.99. A total of 3,454 genes were declared as differentially expressed genes (DEGs) in at least one of the 14 defined comparisons (Additional file 6: Table S5). We found that the fraction of DEGs was significantly higher among genes that were polymorphic (in the sense that they were assigned at least one SNP, in the gene body or a regulatory region, by the VEP tool) than among non-polymorphic ones (Additional file 1: Fig. S2). Therefore, to reduce the influence of introgressions on the results, in the analysis of gene expression that follows we restricted the set of DEGs to the ones that were not polymorphic; however, we retained in the analysis genes in the probable deletion region around the *sdw1* gene. This filtering provided 3,127 DEGs.

**Fig. 5:**
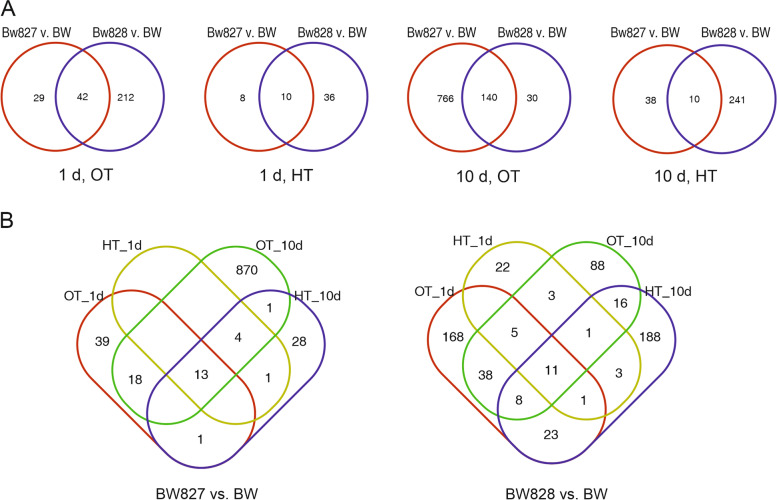
Numbers of differentially expressed genes (DEGs) in two NILs (BW827, BW828) relative to Bowman (BW); **A** DEGs specific and common to NILs in four experimental variants, **B** DEGs specific and common to four experimental variants for two NILs

The DEGs relative to BW were more numerous for BW828 than for BW827 in all experimental variants except at 10 d under OT, where the number of DEGs was highest (Table 2). DEGs were most genotype-specific at 10 d under HT (Fig. 5A). In the four experimental variants, DEGs common to both NILs had the same direction of expression change. The number of DEGs in BW827 vs. BW was larger under OT conditions than under HT conditions at both time points. For both NILs, the DEGs relative to BW were somewhat experimentally variant-specific (Fig. 5B). Considering DEGs observed at different temperatures, for BW828 at 10 d, a large number (24) of common DEGs reacted in different directions. For BW827 under HT, all 18 DEGs at 1 d were also DEGs at 10 d (with the same trend of regulation). However, for BW828 out of 46 DEGs at 1 d, HT, only 16 repeated at 10 d (1 of them with a different trend).

**Table 2:** Numbers of DEGs in comparisons between genotypes and between HT and OT. DEGs in comparisons of genotypes for 4 experimental variants (|log_2_(FC)| > 2, corrected *P* value < 0.05)

Genotype	Regulation	1 d		10 d	
		OT	HT	OT	HT
BW827 v. BW	Down	35	14	85	20
	Up	36	4	821	28
	Total	71	18	906	48
BW828 v. BW	Down	88	17	27	234
	Up	166	29	143	17
	Total	254	46	170	251

**Fig. 6:**
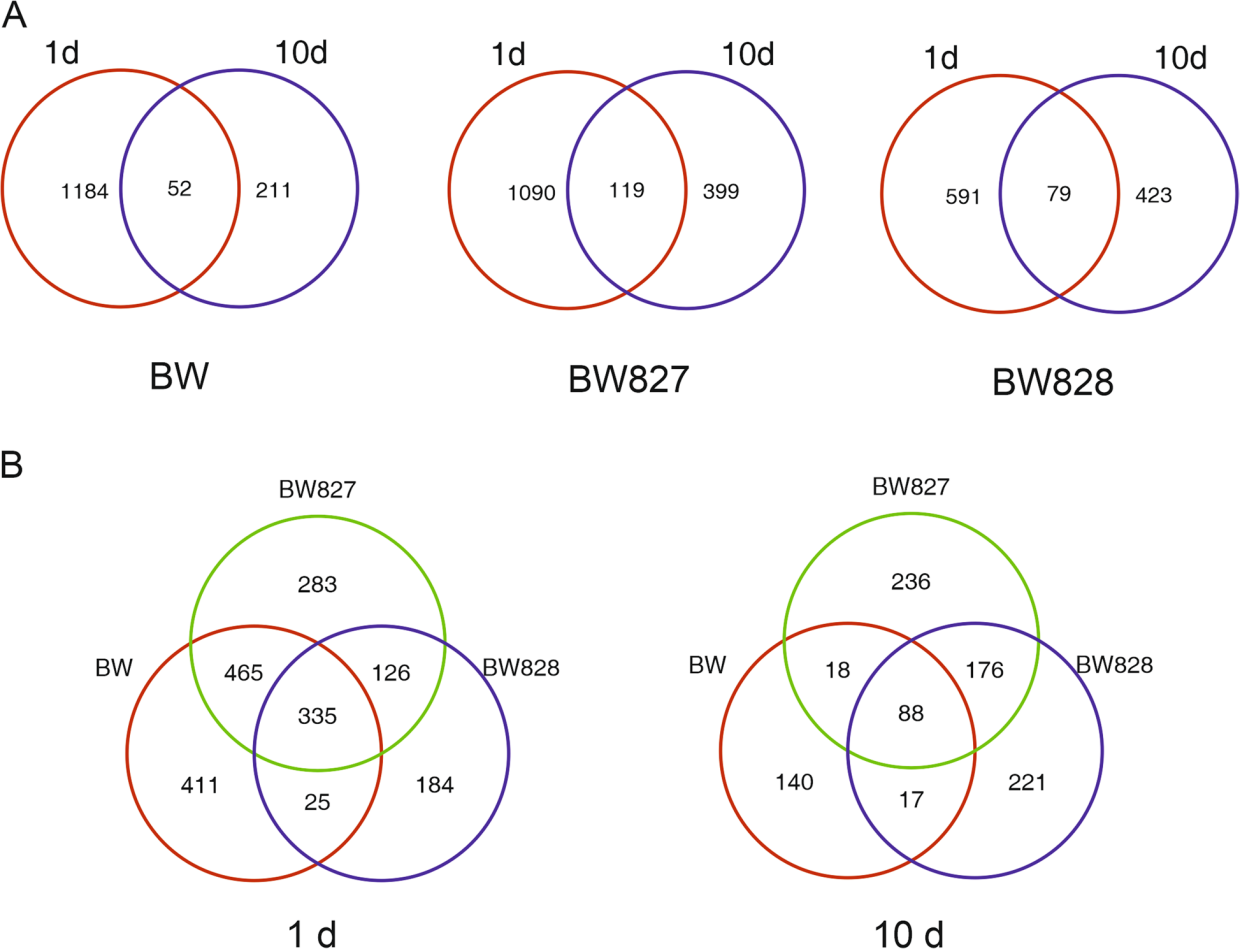
Numbers of differentially expressed genes (DEGs) under elevated temperature (HT) relative to optimal conditions (OT); **A** DEGs specific and common to two time points for three genotypes, **B** DEGs specific and common to three genotypes at two time points

The number of DEGs at HT relative to OT was larger for all genotypes at 1 d than at 10 d (Table 3). The DEGs at HT relative to OT largely changed over time, with a fraction of common DEGs of 4,17% for BW, 7,40% for BW827, and 7.23% for BW828 (Table 3, Fig. 6A); several common DEGs changed the direction of response between 1 and 10 d. Considering different genotypes at a fixed time point, the DEGs were less genotype-specific at 1 d (18.32% of common DEGs) than at 10 d (9.82% of common DEGs) (Table 3, Fig. 6B). The majority of DEGs common for genotype pairs had the same direction of the reaction.

**Table 3:** Numbers of DEGs in comparisons between genotypes and between HT and OT. DEGs in comparisons of variants HT v. OT for three genotypes at two time points (|log_2_(FC)| > 3, corrected *P* value < 0.01)

Genotype	Regulation	Number of DEGs		
		1 d	10 d	Common between 1 d and 10 d (different direction)
BW	Down	1001	28	52 (24)
	Up	235	235	
	Total	1236	263	
BW827	Down	808	314	119 (21)
	Up	401	204	
	Total	1209	518	
BW828	Down	466	317	79 (23)
	Up	204	185	
	Total	670	502

In addition to pairwise contrasts, we also investigated differential responses to elevated temperatures by testing the significance of the interaction-type contrasts (i.e., differences between HT-OT effects in NILs vs. BW) at both time points. At 1 d, differentially reacting genes (DRGs) were much more numerous in the comparison of BW828 vs. BW (Table 4). More genes expressed a differential reaction at 10 d.

**Table 4:** Numbers of DEGs in comparisons between genotypes and between HT and OT. Genes with different reaction to HT in comparison of genotypes NILs v. BW (|log_2_(FC)| > 4, corrected *P* value < 0.01)

Comparison	Number of DRGs		
	1 d	10 d	Common to 1 d and 10 d
BW827 v. BW	3	218	0
BW828 v. BW	211	271	10
Common DRGs	3	116	

Finally, interaction contrasts were tested to compare the temperature effects between time points for all genotypes. More DRGs were observed for BW than for BW827 or BW828 (Table 5). We searched for DRGs that showed significant DEG status with the direction of reaction changing over time (a subset of DRGs). On this basis, 24 genes in BW, 21 in BW827, and 23 in BW828 were identified (Table 3), whose expression was inverted during the growth period under heat treatment relative to optimal conditions (Additional file 7: Table S6). For BW, more DRGs (≈84%) initially exhibited reduced expression under HT and then enhanced expression with prolonged plant exposure to elevated temperatures. The opposite situation was observed for NILs; at 1 d, approximately 33 and 26% of genes were downregulated in BW827 and BW828, under HT, respectively.

**Table 5:** Numbers of DEGs in comparisons between genotypes and between HT and OT. Genes with different reaction to HT in comparison of timepoints 1 d v. 10 d (|log_2_(FC)| > 4, corrected *P* value < 0.01)

Genotype	Number of DRGs
BW	1395
BW827	558
BW828	578
Common DRGs Common to BW, BW827 Common to BW, BW828 Common to BW827, BW828	157 277 578 274

The sets of DEGs and DRGs showed in Tables 2, 3, 4 and 5 were functionally interpreted using Gene Ontology terms enrichment analysis (Additional file 8: Table S7).

In the sets of DEGs between genotypes, 16 GO terms were found to be overrepresented; the largest number of genes was associated with ‘oxidation-reduction’, ‘oxidoreductase activity’, ‘DNA-binding transcription factor activity’, and ‘transcription regulator activity’ (Additional file 8: Table S7A).

In the sets of DEGs between HT and OT, terms related to ‘DNA replication’ were particularly overrepresented at 1 d. More overrepresented terms were found at 10 d in BW827, such as ‘chromatin assembly’, ‘nucleosome organization’, ‘photosynthesis’, ‘response to water’, and ‘response to oxygen-containing compound’. Two terms, ‘oxidation-reduction’ and ‘photosynthesis, light harvesting’, in BW828 (10 d) were enriched within ‘GO biological process’ (Additional file 8: Table S7B).

In the sets of genes responding differentially to heat at different time points, overrepresented GO terms were most numerous for BW827 (terms related to DNA replication, negative regulation of various processes, e.g., ‘hydrolase activity’, ‘proteolysis’, and ‘photosynthesis light-harvesting’) (Additional file 8: Table S7C). Different GO terms were overrepresented for BW828 (‘antibiotic processes’, ‘hydrogen peroxide processes’, ‘oxidation-reduction’, and ‘reactive oxygen species metabolic process’). Fewer terms were found for BW. Interestingly, DRGs assigned to the terms ‘cofactor binding’ and ‘tetrapyrrole binding’ were significantly enriched in both NILs in the comparison between 1 d and 10 d (Additional file 8: Table S7C).

### Genes related to gibberellin and heat

**Fig. 7:**
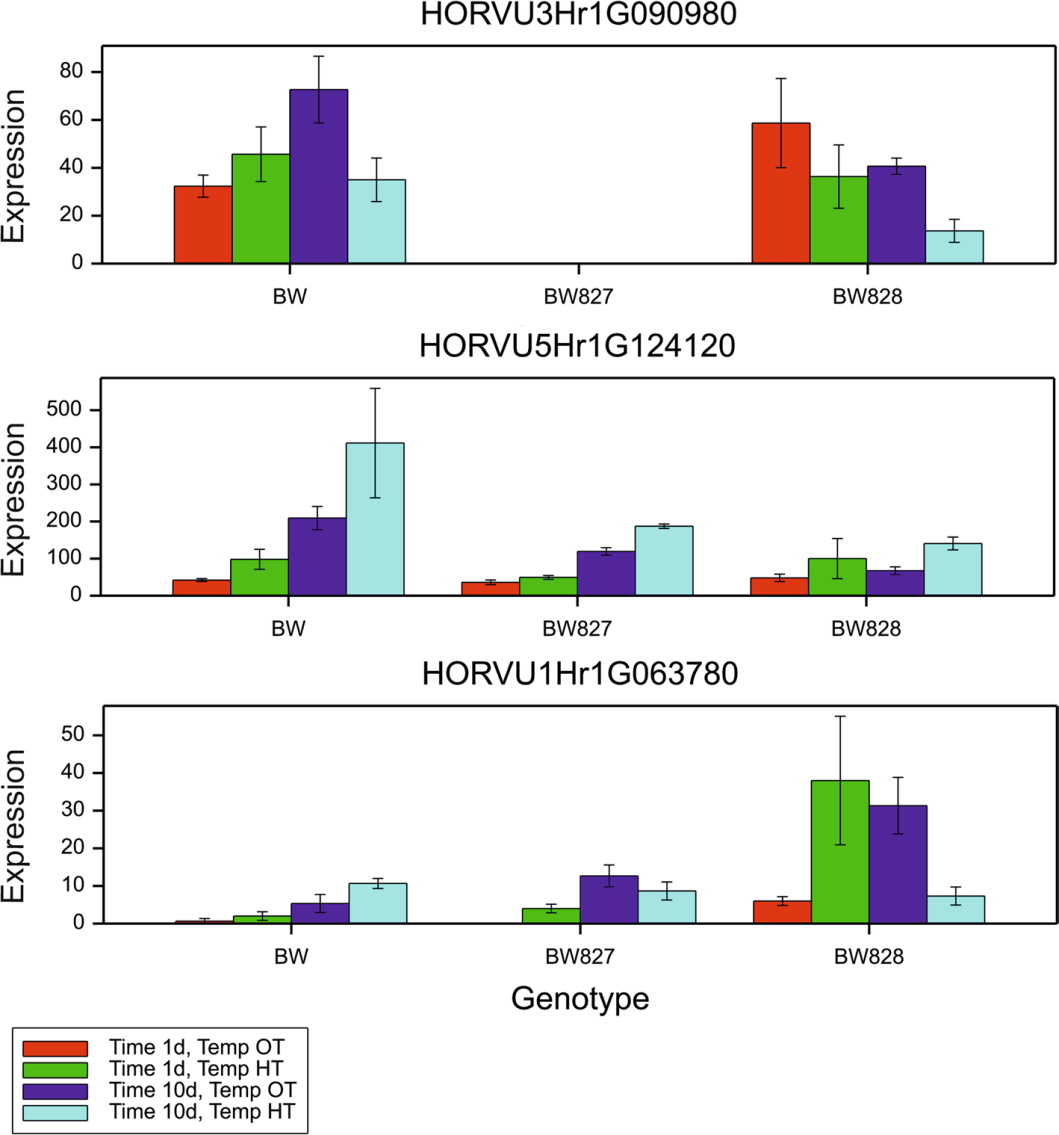
Expression (number of mapped reads) for *sdw1* and its paralogues in barley. Bar = s.e.m. based on 3 biological replications

GO terms related to gibberellin metabolism and signaling were identified (Table 6), and the corresponding 53 GA-related genes are marked in Additional file 6: Table S5. Of these, eight were DEGs in at least one comparison (Additional file 9: Table S8); all were downregulated under HT, mainly at 1 d, and five were found in BW. The expression of one gene, HORVU1Hr1G086810 (GA2-oxidase activity), decreased in all genotypes under HT at 1 d. Comparison between genotypes revealed one other DEG, HORVU1Hr1G063780, encoding GA 20-oxidase (a paralogue of *sdw1/denso*), upregulated in BW828 vs. BW at 1 d (HT) and 10 d (OT) (Fig. 7). No significant expression changes in HORVU3Hr1G090980 (*HvGA20ox2*, *sdw1/denso*) were observed between the OT and HT groups. Based on the DRG analysis, five gibberellin-related genes (HORVU2Hr1G119610, HORVU1Hr1G063780, HORVU1Hr1G086710, HORVU1Hr1G086810, and HORVU0Hr1G018970) showed significant differences in response to HT between 1 d and 10 d. One of them, HORVU1Hr1G086810, positively changed the expression effect over time in all genotypes, i.e., it was downregulated under HT at 1 d and upregulated (but not significantly) at 10 d in all genotypes (Additional file 6: Table S5). The HORVU1Hr1G063780 mentioned above, a paralogue of *sdw1/denso*, changed the effect significantly and negatively only in BW828 over time (downregulation under HT at 10 d relative to upregulation at 1 d). Another paralogue of *sdw1/denso*, HORVU5Hr1G124120 (*HvGA20ox1*), did not show any significant changes in expression (Additional file 6: Table S5, Fig. 7).

**Table 6:** GO terms used to select gibberellin- and heat-related genes

Gibberellin/Heat	GO term ID	GO term name
Gibberellin	GO:1905200	gibberellic acid transmembrane transport
	GO:0010336	gibberellic acid homeostasis
	GO:0009740	gibberellic acid mediated signaling pathway
	GO:0009728	detection of gibberellic acid stimulus
	GO:0009937	regulation of gibberellic acid mediated signaling pathway
	GO:0042388	gibberellic acid mediated signaling pathway, G-alpha-dependent
	GO:0042390	gibberellic acid mediated signaling pathway, G-alpha-independent
	GO:0009939	positive regulation of gibberellic acid mediated signaling pathway
	GO:0009938	negative regulation of gibberellic acid mediated signaling pathway
	GO:0045487	gibberellin catabolic process
	GO:0009686	gibberellin biosynthetic process
	GO:0009685	gibberellin metabolic process
	GO:0010331	gibberellin binding
	GO:0010373	negative regulation of gibberellin biosynthetic process
	GO:0010372	positive regulation of gibberellin biosynthetic process
	GO:0010371	regulation of gibberellin biosynthetic process
	GO:0071370	cellular response to gibberellin stimulus
	GO:0009739	response to gibberellin
Heat	GO:0009408	response to heat
	GO:0031072	heat shock protein binding
	GO:0034605	cellular response to heat
	GO:0010286	heat acclimation

Next, 137 heat-related genes were identified using the GO terms listed in Table 6. Twenty-seven of them were DEGs in at least one comparison (Additional file 9: Table S8). In comparing NILs with BW, five were downregulated and nine were upregulated, most of them in BW828. Five were downregulated and sixteen were upregulated under HT, mostly in BW and BW827, especially at 10 d. Expression of HORVU4Hr1G089090 (HSP70, heat shock protein) decreased in the comparison between BW827 and BW at 10 d (OT) and increased in the temperature comparison between BW827 and BW828 at the second time point (10 d). In contrast, HORVU5Hr1G068320 (HSF, heat shock factor) expression was increased in BW827 vs. BW at 10 d (OT) and decreased in HT vs. OT for BW827 at 1 d. The most significant increase in transcript abundance was observed for HORVU3Hr1G020490 in the comparison between BW828 and BW at 10 d (OT) and for HORVU3Hr1G020500 in the temperature comparison for BW at 10 d (Additional file 6: Table S5). The most substantial negative temperature effect was observed for HORVU6Hr1G094830 in BW and BW828 at 10 d. All these genes are heat shock proteins. Seven heat-related genes expressed differences in response (DRGs) to HT between 1 d and 10 d. One of them, HORVU3Hr1G069590, another heat-shock transcription factor, responded significantly in BW and BW827 over time, i.e., downregulation under HT at 1 d and upregulation at 10 d.

### Phenotypic and physiological characterization

Principal component biplots constructed for post-harvest traits showed a relative similarity of NILs and distinctness of BW (Additional file 1: Fig. S3A). ANOVA revealed significant mean differences between genotypes (Additional file 10: Table S9A). As expected, BW827 and BW828 were, on average, shorter than BW (T3, T8) and had shorter peduncles (T4, T9) and internal internodes (T6, T11) (Additional file 1: Fig. S3B). Differences in basal internodes (T5, T10) were less pronounced; however, the main stem basal internode (T5) was significantly longer in BW828 than in BW827. The number of internodes in the lateral stems (T12) was lower in BW827 and BW828 than in BW. BW827 and BW828 had shorter spikes (T13, T17) with fewer spikelets (T14 and T18) than BW; however, the average number of grains (T15 and T19) was similar in all genotypes. BW828 was characterized by a lower grain weight in the main spike (T16) than the other two genotypes, while a lower grain weight in lateral spikes (T20) was observed for BW827 relative to BW. Grain weight per plant (T21) was similar in NILs but lower than that in BW. In addition, the highest 1,000-grain weight (T22) was observed for BW. Differences between genotypes were not significant for tiller number (T1 and T2). The differences in phenology started to be significant at the flag leaf stage (T24). BW827 and BW828 achieved heading and full maturity (T26) significantly later than BW (by 1–4 days).

Concerning the second source of variation, i.e., the influence of temperature on phenotypic traits, ANOVA (Additional file 10: Table S9A) revealed non-significant or weak negative treatment (HT-OT) effects on internode number (T7, T12). The mean treatment effects were significant and positive for tiller number (T1, T2) and basal internode length (T5, T10); in contrast, they were significant and negative for the rest of the phenotypic and phenological traits. Genotype-specific effects of temperature were observed for length measurements of stems (T3, T8) and internal internodes (T11); the effects were smaller for NILs than for BW. Another genotype-specific effect of temperature on main spike length (T13) was significant only for BW827.

Significant differences in photosynthetic parameters between genotypes were observed for ABS/CS, TR/CS, and ET/CS, with the highest values for BW827 (Additional file 10: Table S9B, Additional file 1: Fig. S4A). The temperature effect was significant for ABS/CS, DI/CS (with lower values under HT), and ET/CS (with higher values under HT). Genotype-specific treatment effects were observed for RC/CS, where the HT effect was negative for BW827 and positive for BW828.

The mean levels of flavonols and anthocyanins were highest in BW828 (Additional file 10: Table S9B, Additional file 1: Fig. S4B). The treatment effect was significant for all pigments; negative for chlorophyll and anthocyanins and positive for flavonols. Genotype-specific treatment effects were observed for flavonols; they were positive for all genotypes but of different magnitudes (smallest for BW827, largest for BW828).

ANOVA for RWC observed at two time points revealed significant differences between genotypes (*P* = 0.047), with the lowest average RWC level in BW827 (Additional file 1: Fig. S4C), between time points (*P* < 0.001), with the lowest level at 10 d, and between treatments (*P* < 0.001), with RWC lower under HT. The treatment effects were genotype-specific; the effect of HT was largest for BW828 (-6.86) and smallest for BW827 (-3.25).

## Discussion

Sanger sequencing confirmed a total deletion of the *sdw1/denso* candidate gene in BW827 (*sdw1.a*), a 7-bp deletion, and a G/A substitution in BW828 [34, 35]. SNP and gene expression data obtained for BW827 indicated a deletion of 0.555 Mb around the *sdw1/denso* locus, which contained genes related to, i.a., methylation, phosphorylation/kinase activity, regulation of transcription, oxidoreductase, and transporter activity. Deletion of this fragment may significantly affect important regulatory-related functions in BW827. Our genome-wide genotyping, which was more extensive than previously performed in a ‘Bowman’-derived NILs collection [32] using Illumina Golden Gate BOPA1 and BOPA2 assays, providing almost 20 times more SNPs, revealed that the BW827 genome was much nearer to BW than BW828. This could be a consequence of the lower number of backcross cycles (only four) performed to develop BW828 relative to the BW827 breeding process (seven or more BC rounds) [32]. This contrasts with the report [36] which claimed that both BW827 and BW828 retained only small-donor introgressions, despite the different numbers of BC cycles for each NIL.

Taking into account the observed degree of polymorphism and different origins of introgressions (from ‘Jotun’ in *sdw1.a* and ‘Valticky’ in *sdw1.d*), it can be assumed that differences between NILs could be linked to the genetic background diversity in addition to the single *sdw1/denso* allele variation. Therefore, to limit the impact of multiple introgressions on differential expression analysis, the polymorphic genes, excluding those located in the *sdw1* region, were filtered out. Such approach intended to highlight the specific effects of 3H introgression carrying *sdw1* on other genes' expression. However, it did not reduce significantly the overall number of detected DEGs. Thus, we assumed that although the pleiotropy or epistasis of genes around the *sdw1* locus may occur, the discussed transcriptomic results cannot be univocally interpreted as an effect of the mentioned 3H introgression. Noteworthy, some removed DEGs can play important regulatory functions. For instance, the gene HORVU3Hr1G088200, encoding WRKY transcription factor, contained SNP with HIGH predicted protein translation effect (i.e., truncation of this protein or loss of function may occur in *sdw1.d* mutants) and was assigned seven other SNPs. Notably, with no SNP in BW relative to BW827, this gene showed the same pattern of negative expression change under early HT in these genotypes, whereas no expression changes in BW828 were observed. Thus, it can be inferred that mutations related to HORVU3Hr1G088200 in BW828 affected gene regulation. WRKY transcription factors coordinate developmental processes and plant responses to biotic and abiotic stresses. They mediate hormonal signal transduction as both negative and positive regulators and have a tissue-specific expression [37, 38]. WRKY TFs are predominantly overexpressed upon stress stimuli to help plants cope with adverse conditions [39]. The gene HORVU3Hr1G088200 is annotated to the negative regulation of the gibberellic acid-mediated signaling pathway and putatively encodes WRKY33 (*HvWRKY33*). In *Arabidopsis thaliana*, high-temperature stress repressed the expression of *AtWRKY33*, which resulted in enhanced activity of *AtWRKY25* and *AtWRKY26*. In turn, overexpression of *AtWRKY25* and *AtWRKY26* increased plant resistance to heat [40]. It cannot be excluded that an analogous mechanism may also exist in barley’s crown tissue in BW and the *sdw1.a* mutant.

Our RNA-seq experiment provided novel data on gene expression in barley crowns since, according to EBI Expression Atlas, no information for this tissue is available there. As expected, the genotype effect on gene expression was smaller than the temperature effect since genetically related, albeit polymorphic, barley forms were used in the present study. The number of DEGs between BW827 and BW was approximately 2–3-fold lower than that between BW828 and BW, with common DEGs reacting in the same direction. The transcriptomic response of BW827 to elevated temperature was more similar to the BW response than that of BW828. Generally, exposure to increased temperature over time induced more changes in gene reactions (DRGs) in BW than in NILs. Some genes showed different directions of expression changes at the two time points. One of them, HORVU2Hr1G012200, which encodes a protein belonging to the calcium-dependent lipid-binding (CaLB domain) family, responded initially negatively to HT, but after prolonged temperature treatment, its expression was enhanced in all genotypes. Such a uniform response can be associated with the conserved role of the mentioned proteins in signaling. CaLB domain interacts with membranes in a Ca^2+^-dependent manner and is involved in signal transduction [41].

Eight GA-related DEGs were detected in our study by referring to the particular classes of genes analyzed. To date, the expression of gibberellin oxidase genes has been reported to be both up- and downregulated in response to high temperature treatment [42]. In general, decreased expression of *GA20ox* and *GA3ox* and increased *GA2ox* expression resulted in GA content reduction in plants exposed to abiotic stress [43]. This was not confirmed in our study, since all nine *GA2ox* DEGs were downregulated in crowns in response to HT. Expression of the *sdw1/denso* candidate gene (*HvGA20ox2*, HORVU3Hr1G090980) was not modified by increased temperature in either the crown of BW or BW828. It was demonstrated by [34] that partial or total loss of function of *HvGA20ox2* was compensated in barley leaves by both *HvGA20ox1* (HORVU5Hr1G124120) and *HvGA20ox3* (HORVU3Hr1G089980) stimulation, wherein the first and the second paralogs were dominant in the ‘Baudin’ (*sdw1.d*) and ‘Jotun’ (*sdw1.a*) mutants, respectively. Similar findings were reported by [31]. However, we did not observe this in the crowns of ‘Bowman’-derived *sdw1* mutants, where *HvGA20ox2* and its above-mentioned paralogs’ expression were not significantly modified relative to the wild type and were not affected by HT. Interestingly, we identified another paralogue (HORVU1Hr1G063780) of *HvGA20ox2* more strongly expressed in BW828 than in BW and BW827. According to [44], it is the ortholog of rice and wheat genes encoding GA20 oxidase-4, and is thus defined as *HvGA20ox4* in barley. It showed significant overexpression in optimal and early high temperature treatment, being reduced within the time course of stress application only in BW828. This indicates that *HvGA20ox4* may partially take over the *HvGA20ox2* role in the GA biosynthesis pathway in the crown of the *sdw1.d* mutant during tillering. However, we did not examine whether the *sdw1* mutation in BW828 resulted in the production of a dysfunctional protein. Nevertheless, a higher expression of the aforementioned paralogous gene did not overcome the GA deficiency symptoms, and we hypothesized that it simply induced longer basal internode formation in BW828 relative to BW827. Of note, the *HvGA20ox4* gene was not considered in the above-mentioned study concentrated on characterization of the *sdw1* gene in barley by [34].

Notably, reduced expression of HORVU1Hr1G086810 in response to early HT was observed in all studied accessions. This gene encodes GA2 oxidase, one of critical enzymes in the gibberellin biosynthesis pathway, leading to the deactivation of GAs. Its overexpression represses plant growth and promotes the dwarf phenotype [23, 24]. In general, a positive effect of high temperature treatment on basal internode tiller production was observed in our study. This, together with evidence of a negative relationship between GA content and tiller number, could indicate that decreased expression of the HORVU1Hr1G086810 gene in the crown might contribute to BW and NIL phenotypes under elevated temperature through tillering stimulation and basal internode elongation, although the final plant height remained lower than in control plants.

Application of elevated temperature induced more changes in the expression of heat-related genes in BW827 (*sdw1.a*) and BW than in BW828 (*sdw1.d*). Downregulation of HORVU4Hr1G089090 in BW827 relative to BW was observed under optimal conditions; however, late temperature treatment affected its expression positively in both NILs. This increased expression was expected because the gene encodes heat shock protein (HSP70). On the other hand, expression of the HORVU5Hr1G068320 gene encoding heat shock factor (HSF) was unexpectedly increased in the *sdw1.a* mutant relative to BW under optimal conditions (10 d, OT) and decreased at an early time point of exposure to the higher temperature (1 d, HT). As indicated by [45], this gene, defined as *HvHsfB1*, contains numerous regulatory elements in the promoter region, including hormone-responsive *cis*-acting elements. This could justify the erratic activity of the gene in our study, since *Hsf* genes are suggested to be engaged not only in stress response but also in plant growth and development. In addition, most of the DRGs whose reactions were significantly modified by prolonged high temperature treatment were annotated as HSP20, HSP70, and HSF. This is not surprising because HSF family proteins are master agents in the induction of response to heat stress, while HSPs, as molecular chaperones, represent a major class of thermoprotective factors [46]. It has been known that HSPs and HSFs interact together and regulate many processes underlying plant responses to environmental stresses. Nonetheless, there is growing evidence that both protein families may also play an important role in overall plant growth and development [45]. Evidence shows that HSPs70/90 are negative regulators of the *HsfA1* gene, which is released from repression and becomes active in response to heat; in this way, it can affect other stress-inducible genes, including TFs (e.g., DREB2A) [46]. Phylogenetic analysis of HSF-coding genes in barley revealed 23 *HvHsf* candidate genes distributed in all chromosomes [45]. Five of them, representing all classes (A–C), were found to be differentially expressed in crown tissue, including *HvHsfA2e* (HORVU5Hr1G094380), *HvHsfA7b* (HORVU7Hr1G087690), *HvHsfB1* (HORVU5Hr1G068320), *HvHsfB2b* (HORVU7Hr1G056820), and *HvHsfC1b* (HORVU3Hr1G069590). Interestingly, DEGs specific to BW828 (*HvHsfA2e*, *HvHsfA7b)* were upregulated under HT at the late time point (10 d); in contrast, those specific to BW827 were downregulated under HT at 1 d (*HvHsfB1, HvHsfC1b*) or 10 d (*HvHsfB2b*). On the other hand, the *HvHsfC1b* gene, also detected in DRGs analysis, reacted identically, from negative to positive regulation status over time, in BW and the *sdw1.a* mutant. This uniform response across genotypes can be explained by the fact that proteins of the HSF family are highly conserved TFs in the plant kingdom [47]. The present study provides novel information on HSF transcriptomes in barley crowns, since no reports on *HvHsf* genes activity in this tissue have been available to date. Merely, a recent report by [45] compared *HvHsf* gene expression in differentially treated shoots and roots of barley, with a large discrepancy between the activity of these genes. Some of the *HvHsf* genes showed no temperature induction (e.g., *HvHsfC1b*), while others were highly expressed (e.g., *HvHsfA2e*). It is worth noting that a great variation in a single gene expression between the shoot and root was observed by [45]. It can be assumed that the crown tissue-specific expression patterns of *HvHsf* genes also exist.

The broader functional interpretation of genes, whose expression was affected by high temperature treatment, revealed more stress-related categories to be enriched in BW827 than in BW828, especially at 10 d. Therefore, we concluded that the late transcriptome response of the *sdw1.a* mutant to high temperature treatment was functionally more concentrated than that of the *sdw1.d* mutant.

Gene Ontology analysis of differentially reacting genes (DRGs) revealed enrichment of divergent functions between the NILs. In BW827, numerous genes controlling DNA replication and negatively regulating various processes, such as ‘peptidase activity’ and ‘proteolysis,’ became active over time under heat exposure. On this basis, it can be assumed that in *sdw1.a* mutant, more progressive degradation of DNA/proteins under prolonged heat treatment might occur. On the other hand, enrichment of DRGs assigned to ‘hydrogen peroxide processes,’ ‘oxidation-reduction,’ and ‘reactive oxygen species metabolic process’ in the *sdw1.d* mutant may suggest its more efficient molecular machinery of adapting to temperature stress. Simultaneously, overrepresentation of the ‘tetrapyrrole binding’ category was observed for both NILs, suggesting that significant temperature-induced detoxification of reactive oxygen species mediated by tetrapyrroles was specific for *sdw1* mutants [48].

Oxidation-reduction is an elementary biological process responsible for cell homeostasis, plant development, and defense [49]. It promotes molecular oxygen to generate reactive oxygen species (ROS), which mediate the modulation of gene expression underlying early stress response [50]. In addition, ROS are considered second messengers in plant signaling and trigger cell apoptosis and oxidative stress responses [51, 52]. The aforementioned DEGs and DRGs assigned to oxidation-reduction processes and associated categories could be expected since barley accessions were subjected to oxidative stress due to heat treatment. However, identification of the enriched oxidation-reduction category within DEGs between genotypes indicated that although the barley forms were genetically related, their oxidative response seemed to be genotype-specific.

In BW827, photosynthesis-related terms were overrepresented in the annotation of genes with altered expression or response to stress over time. It seems surprising since crown tissue is a photosynthetically inactive region [53]. However, in report of [19], who conducted microarray transcriptome analysis of winter barley crowns exposed to chilling, the induction of photosynthetic genes in this tissue was also identified. Similar findings were reported by [54] in relation to low-temperature stressed wheat crowns. We hypothesize that some of the genes annotated to photosynthesis may also play a protective role in plants, which was suggested two decades ago by [55]—this deserves a more detailed investigation.

Phenotyping confirmed the known phenotypic effects of the *sdw1* locus [56, 57]. The decreased plant height of NILs was primarily due to the shortened spikes and internodes, especially through peduncle reduction. This is in contrast to study done by [31], who reported that reduction of two basal internode lengths caused the semi-dwarf phenotype of the *sdw1.e* mutant (Riso no. 9265), whereas peduncle and spike length were relatively similar to those of the wild type. We confirmed decreased grain yield and 1,000-grain weight in both NILs, as seen in the data available in the International Database for Barley Genes and Barley Genetic Stocks (BGS 518, sdw1), although positive effects of the *sdw1* locus on grain yield have also been reported, e.g. [26, 58]. Although a negative correlation between tiller formation and GA content has been suggested [59] and *sdw1* barley plants were described as high-tillering phenotypes [14], the positive effect of *sdw1* on tillering of NILs was not revealed in our experimental layout. Interestingly, BW827 had a lower tiller number and smaller and larger grain weight in lateral and main spikes, respectively, compared with BW828.

Overall, the negative effect of elevated temperature was found for most phenotypic traits and phenology; however, it was positive for tiller number and basal internode length. Despite the promotion of tillering under elevated temperatures, additional shoots were infertile or contained sparsely developed grains, which negatively affected total yield. The largest negative genotype-specific impact of HT was observed for stem length in BW. The temperature treatment was less harmful to the plant height of *sdw1* mutants than BW.

Differences between genotypes were identified for most physiological traits. Altogether, the response of BW828 to HT was distinguished among genotypes by the highest content of all pigments under stress and the most significant late reduction in RWC. It may be that the photosynthetic apparatus of BW828 is protected explicitly against high temperatures owing to the observed positive effect of HT on the density of PS II reaction centers (RC/CS), which was negative for BW827. Flavonoids are known to play important antioxidant and protective roles in plants exposed to abiotic stresses [60], and as expected, elevated temperatures increased flavonol content in leaves across genotypes, mostly in BW828. However, anthocyanins accumulation was decreased since stress severity was not sufficient to enhance their synthesis. In report [61] authors attempted to explain the high ambient temperature repression of anthocyanin biosynthesis in Arabidopsis mutants by alluding to HY5 degradation (long hypocotyl5), which leads to enhanced and reduced expression of anthocyanin negative regulators and biosynthesis genes, respectively.

## Conclusions

In conclusion, considering that ‘Bowman’-derived *sdw1* NILs were proven to be genetically much more distant than their phenotypic similarity would indicate, we claim that transcriptomic genotype-specific heat responses of *sdw1* mutants measured in their crown tissue resulted from wider genetic background diversity than from variation of *sdw1* alleles or multiple gene deletion. The most contrasting response to elevated temperature between NILs was identified for *HvHsf* genes. Differences in the expressional reaction of genes to heat in different *sdw1* mutants as well as changing regulation status of genes over time, found to be independent of the polymorphism, could be further explained by in-depth studies of the regulatory factors acting in the studied system.

## Methods

### Plant material

Plant material included two-rowed spring barley (*Hordeum vulgare* L.) of the ‘Bowman’ cultivar (BW, wild type) and its two near-isogenic lines (NILs), BW827 and BW828, which carry *sdw1.a* and *sdw1.d* mutations, respectively (Fig. 1), obtained by X-ray treatment in varieties ‘Jotun’ and ‘Valticky’. BW827 and BW828 were developed by recurrent backcrossing of the mutants to ‘Bowman’ [32]. Seeds of used plant material were obtained from the Nordic Genetic Resource Center (NordGen).

### Sequencing and genotyping

Sequencing of the *HvGA20ox2* gene (*sdw1*; HORVU3Hr1G090980) and its upstream region was performed using the Sanger method in three biological replicates. The results were analyzed using Codon Code Aligner software. First, for each accession, the consensus sequence of the *HvGA20ox* gene was assembled. The three sequences were then aligned to gain insight into the polymorphisms among the studied accessions. All the identified SNPs were confirmed by independent sequencing.

The overall genetic composition of the barley forms was investigated using three approaches: single nucleotide polymorphism (SNP) calling from RNA-seq data (described below), genotyping by sequencing (GBS) with SNP detection conducted by LGC Genomics GmbH (Berlin, Germany), and genotyping using a 50k Illumina Infinium iSelect SNP array [62] conducted by TraitGenetics GmbH (Gatersleben, Germany). GBS was conducted by applying the ddRAD-Seq protocol described by [63], using the Illumina NextSeq 500 platform with a sequencing depth of 3 M read pairs per sample, and SNP calling performed by the service provider’s pipeline. To perform GBS and SNP chip assays, genomic DNA was extracted from 2-week-old leaves using the Wizard® Genomic DNA Isolation Kit (Promega, Madison, WI, USA) according to the manufacturer’s instructions. DNA quality and concentration were assessed using a NanoDrop2000 spectrophotometer (Thermo Fisher Scientific, Waltham, CA, USA) at ratios of >1.8 for 260/280 and 260/230. DNA samples were diluted to 50 ng/μL using molecular biology-grade water (Merck, Darmstadt, Germany). Frozen DNA solutions (20 μL) were submitted for genotyping.

### Experimentation

Plants were grown in pots (H-LSR 4.5 L; 21 cm in diameter and 20 cm in height) filled with a mixture of loamy soil and peat (3:1, *w*/*w*) under controlled conditions (60% air humidity, 234 μmol m^−2^ s^−1^ PAR irradiance; Apollo 8 LED Grow Light). The number of pots was set to provide material for all studies. Eight seeds per pot were sown, and after germination, the number of plants was reduced to five. Two temperature regimes were applied: (i) optimal (control) temperature (OT) of 8/16 °C (night/day) from sowing to the end of tillering and then 12/20 °C to maturity; (ii) elevated temperature (HT) of 28 °C from sowing to the end of tillering and then 12/20 °C as in the control. An 8/16 h (dark/light) photoperiod was maintained, while soil moisture was maintained above 70% field water capacity, controlled by the daily weighing of each pot.

### Transcriptomics

Barley crown tissue (Fig. 4) was sampled for gene expression analysis using mRNA-seq in three biological replications at two time points: 1 d, tillering stage (23-26 of BBCH code), and 10 d, ten days after the 1 d. Each replication consisted of crown samples collected from three plants per pot. Total RNA was extracted using TRI Reagent® RT (Molecular Research Center, Inc., Cincinnati, OH, USA) according to the manufacturer’s protocol and treated with DNase I during RNA purification. The quality and quantity of RNA were verified using a NanoDrop 2000 spectrophotometer (Thermo Fisher Scientific) using the following criteria: 2.0 for 260/280 and 260/230 ratios. RNA integrity number (RIN) of samples sufficient for sequencing (≥ 8) was confirmed using an Experion™ electrophoresis station (Bio-Rad Laboratories, Hercules, CA, USA). cDNA library construction (TruSeq stranded mRNA) and sequencing were conducted by Macrogen Inc. (Seoul, Republic of Korea) using an Illumina NovaSeq6000 platform with a 100 bp paired-end configuration and 35.7–51.9 M reads per sample.

### Phenotyping and physiological characteristic

Mature plants were harvested manually and scored for traits associated with the plant structure and yield potential: spike and grain characteristics, features of the peduncles and internodes of primary (main) and secondary (lateral) stems, and timing of particular developmental stages (for the list of observed traits, see Additional file 10: Table S9).

The physiological examination included (i) photosynthetic parameters measured using a PocketPea fluorimeter (Hansatech Instruments, Norfolk, England): ABS/CS, absorption energy flux per CS; TR/CS, trapped energy flux per CS; RC/CS, density of RCs (Q_A_^-^ reducing PSII reaction centers); ET/CS, electron transport flux per CS; DI/CS, dissipation energy flux per CS; (ii) photosynthetic pigments content measured using a Dualex meter (Force-A, Orsay, France): index of chlorophyll, flavonols, and anthocyanins; (iii) RWC (%), relative water content [64]. A 30-minute dark adaptation period was adopted, then leaves were immediately exposed to a pulse of saturating light at an intensity of 3,500 μmol m^-2^ s^-1^ with a wavelength of 627 nm. Physiological traits were measured on the second leaf of plants at 1 d under OT and HT; RWC was additionally analyzed at 10 d.

Phenotyping and physiological studies were conducted in three biological replicates, with each replicate consisting of five plants from one pot and represented by average trait values.

### Data analysis

Statistical analyses of phenotypic and physiological data and visualization of results not attributed below to other software were performed using Genstat 19 [65]. Principal component biplots were created after centering and normalizing the data. Analysis of variance was performed in a model containing fixed effects of genotype (G), temperature treatment (T), and G × T interaction.

The IBSC_v2 *Hordeum vulgare* (Ensembl Plants rel. 41) genome assembly was used as a reference for SNP and gene expression analyses. After removing adapter-related sequences and quality trimming using AdapterRemoval ver 2.1.7 [66] (parameters: –minquality 20, –minlenght 50), mRNA-seq reads were mapped in the reference using TopHat ver. 2.1.1 [67] (parameters: maximum no. of mismatches = 1, --no-mixed, --library-type fr-firststrand, --no-discordant); the mapping efficiency was 70-86%. Reads aligned to annotated transcripts were counted using the featureCounts function in Bioconductor, R 3.6.1 (Rsubread library [68];) and the resulting data were subjected to differential expression analysis in Deseq2 [69]. Differentially expressed genes (DEGs, differing in expression between two experimental variants) and differentially reacting genes (DRGs, differing in reaction to treatment between two genotypes or two time points) were found among the genes characterized by a mean expression of at least 10 units (estimated in Deseq2). Gene Ontology terms enrichment analysis was performed using the hypergeometric test, with computation of family-wise error rates (FWER) using the GOfuncR library in Bioconductor [70]. SNP calling in mRNA-seq data pooled from three biological replications for three genotypes, in optimal conditions at 1 d, was performed using the samtools/bcftools pipeline [71] (filtering parameters: %QUAL > 20, MAF > 0.10, DP > 40). Venn diagrams were drawn using the ‘venn’ package in R. SNP protein translation effects were predicted using the VEP tool (Ensembl Plants [72];). SNP visualizations were performed using IGV [73].

## Supplementary Information

**Additional file 1: Figure S1.** Barley gene HORVU3Hr1G090980 (*sdw1*), with isoforms and polymorphisms identified by Sanger sequencing in BW828. Genotypes are given in the order BW_BW827_BW828. (Visualization in IGV, software.broadinstitute.org). A neighboring gene HORVU3Hr1G090970, with SNPs found by genotyping and RNA-seq, is also shown. **Figure S2.** Fractions of differentially expressed genes (DEGs) among polymorphic and non-polymorphic genes. (A) DEGs found in the comparison between Bowman and BW827, (B) DEGs found in the comparison between Bowman and BW828. *P* values obtained in the chi-square test for homogeneity of fractions among three groups of polymorphic and non-polymorphic genes. **Figure S3.** (A) Biplots for phenotypic observations under OT and HT, (B) Mean values (with std. errors) of phenotypic traits for three barley genotypes observed under OT and HT. **Figure S4.** (A) Mean values of photosynthetic parameters, (B) Mean values of pigments, (C) RWC, mean values for genotypes under HT, OT at time points 1 d and 10 d.

**Additional file 2: Table S1.** Genes in deletion region in BW827.

**Additional file 3: Table S2.** SNP of 3 types observed in 3 genotypes.

**Additional file 4: Table S3.** Differences in SNP readings between protocols.

**Additional file 5: Table S4.** GO overrepresentation in sets of genes with SNPs.

**Additional file 6: Table S5.** Results of differential expression analysis.

**Additional file 7: Table S6.** DEGs with opposite sign of HT-OT effect between 1 d and 10 d.

**Additional file 8: Table S7.** GO overrepresentation for sets of DEGs and DRGs.

**Additional file 9: Table S8.** Gibberellin and heat related genes.

**Additional file 10: Table S9.** Phenotypic and physiological traits, with analysis.

## Data Availability

All data generated and analysed during this study are included in the published article and its supplementary information files. Additionally, RNA-seq data used in this paper are available in the ArrayExpress repository, accession number E-MTAB-10789 (https://www.ebi.ac.uk/arrayexpress/experiments/E-MTAB-10789) – access open after publication. Public, open access database EnsemblPlants (https://plants.ensembl.org) was also used for raw data processing.

## Abbreviations

1 d: Time point, tillering stage (23-26 of BBCH code)
10 d: Time point, ten days after the 1 d
BC: Backcrossing
BW: cv. Bowman
CaLB domain: Calcium-dependent lipid-binding domain
DEGs: Differentially expressed genes
DRGs: Differentially reacting genes
GAs: Gibberellins
GBS: Genotyping by sequencing
GO: Gene Ontology
HSF: Heat shock factor
HSPs: Heat shock proteins
HT: Heat treatment
NGS: Next generation sequencing
NILs: Near-isogenic lines
OT: Optimal (control) temperature
ROS: Reactive oxygen species
RWC: Relative water content
SNP: Single nucleotide polymorphism
TFs: Transcription factors
