# Supplementary figures and images for: High-throughput sequencing data revealed genotype-specific changes evoked by heat stress in crown tissue of barley sdw1 near-isogenic lines
Source: BMC Genomics. 2022 Mar 4;23:177. doi: 10.1186/s12864-022-08410-1 (PMC8897901; doi:10.1186/s12864-022-08410-1)

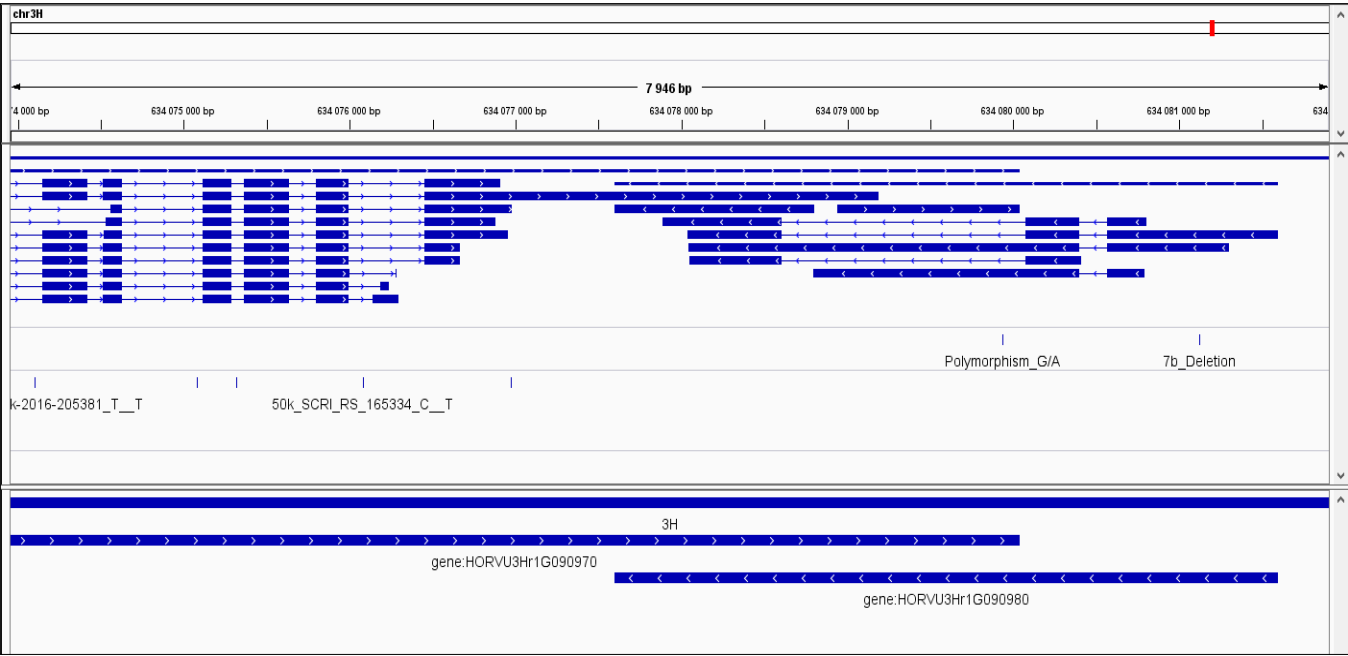

Figure S1

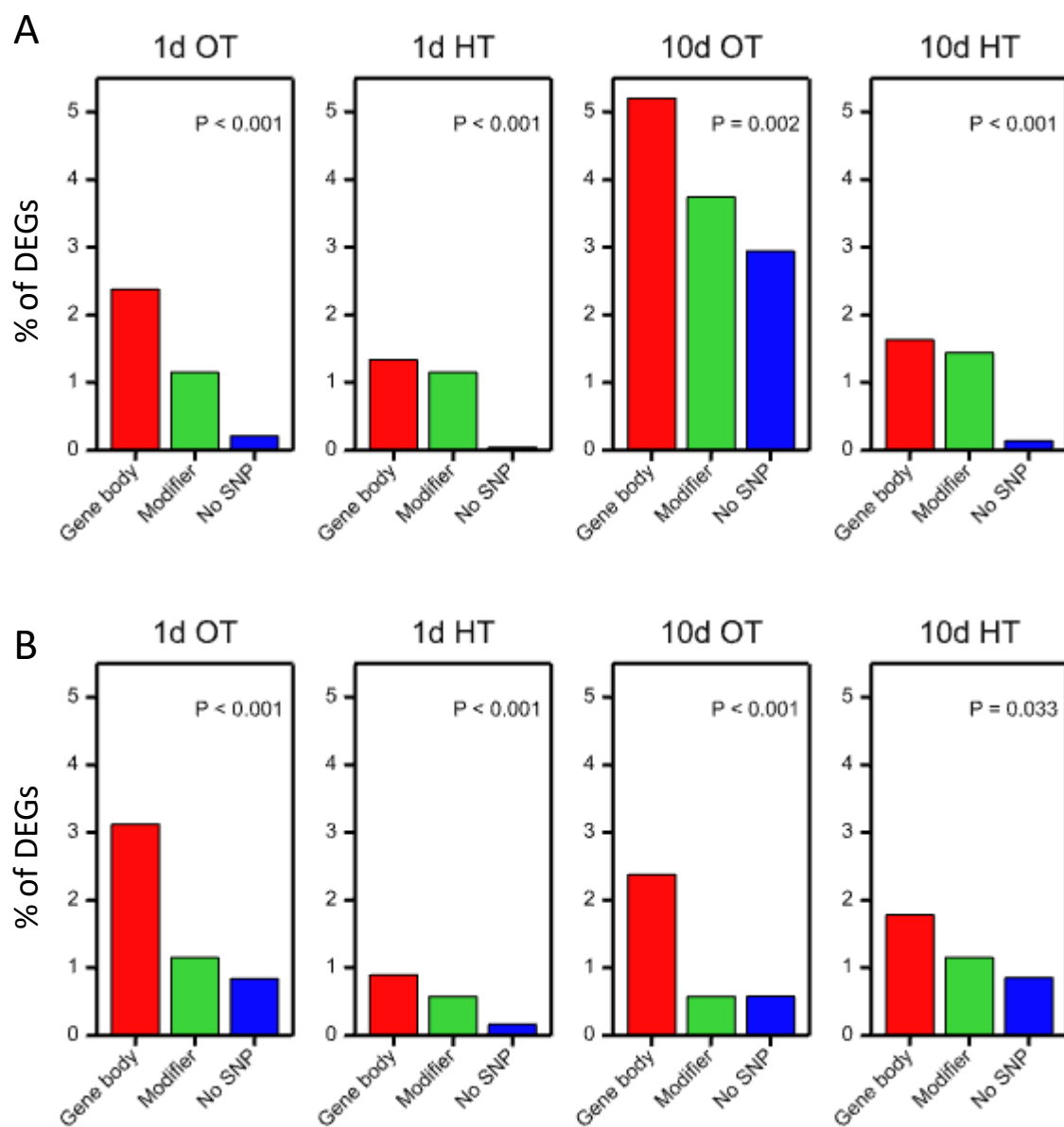

Figure S2

A

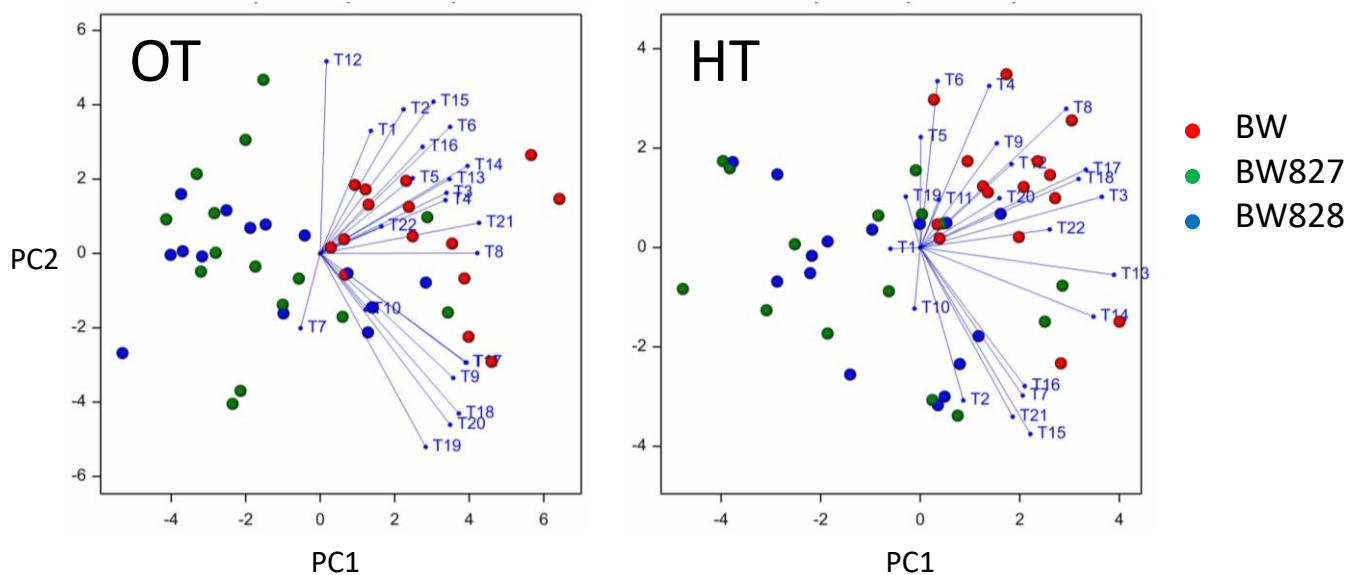

B

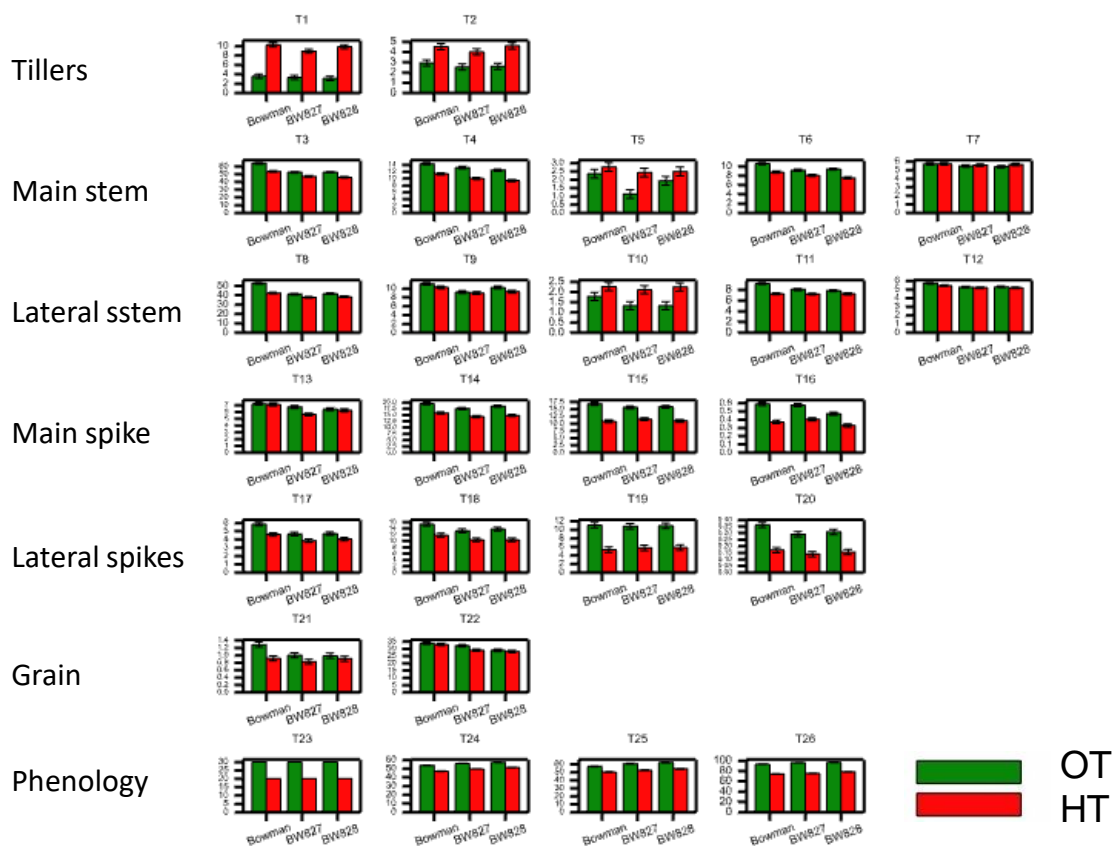

Figure S3

A

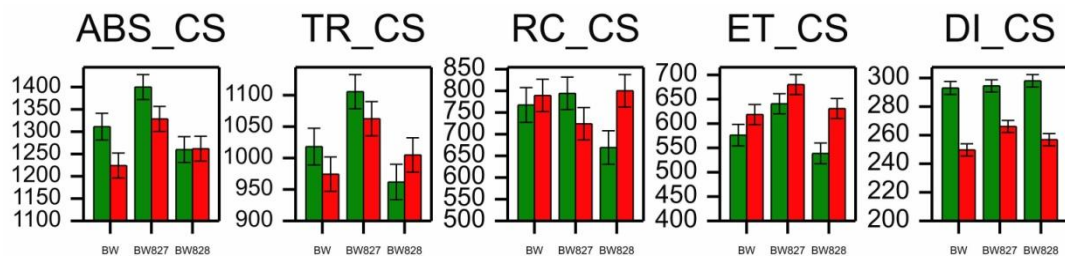

B

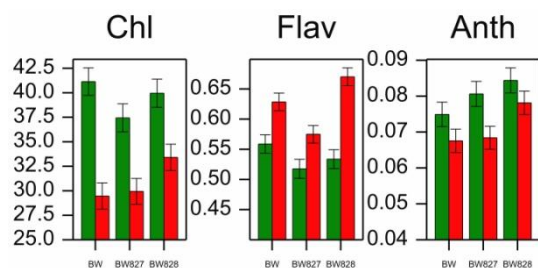

C

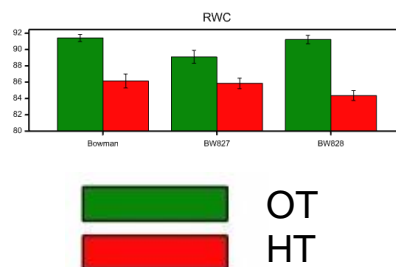

Figure S4

Supplement: Supplementary file 1 — Additional file 1: Figure S1. Barley gene HORVU3Hr1G090980 (sdw1), with isoforms and polymorphisms identified by Sanger sequencing in BW828. Genotypes are given in the order BW_BW827_BW828. (Visualization in IGV, software.broadinstitute.org). A neighboring gene HORVU3Hr1G090970, with SNPs found by genotyping and RNA-seq, is also shown. Figure S2. Fractions of differentially expressed genes (DEGs) among polymorphic and non-polymorphic genes. (A) DEGs found in the comparison between Bowman and BW827, (B) DEGs found in the comparison between Bowman and BW828. P values obtained in the chi-square test for homogeneity of fractions among three groups of polymorphic and non-polymorphic genes. Figure S3. (A) Biplots for phenotypic observations under OT and HT, (B) Mean values (with std. errors) of phenotypic traits for three barley genotypes observed under OT and HT. Figure S4. (A) Mean values of photosynthetic parameters, (B) Mean values of pigments, (C) RWC, mean values for genotypes under HT, OT at time points 1 d and 10 d. [file 12864_2022_8410_MOESM1_ESM.pdf]
